# Clinico-pathological and renal morphological findings in dogs naturally infected with *Dirofilaria immitis*

**DOI:** 10.1186/s12917-026-05535-3

**Published:** 2026-05-02

**Authors:** Korinna É. Szabó, Luca Aresu, Linda Müller, Eszter Szilágyi, Fruzsina A. Falus, Nándor Balogh, Márton Boros, Tibor Papp, Ferenc Manczur

**Affiliations:** 1 Department of Internal Medicine, University of Veterinary Medicine, Budapest, Hungary; 2 Department of Veterinary Sciences, University of Turin, Grugliasco, Italy; 3 European Veterinary Renal Pathology Service, MyLav Laboratory, Milan, Italy; 4 Department of Obstetrics and Food Animal Medicine Clinic, University of Veterinary Medicine, Budapest, Hungary; 5 Praxislab Ltd., Budapest, Hungary; 6 HUN-REN Veterinary Medical Research Institute, Budapest, Hungary

**Keywords:** *Dirofilaria repens*, Dog, Renal histopathology

## Abstract

**Background:**

*Dirofilaria repens* is a mosquito-borne filarial nematode that causes subcutaneous dirofilariasis in dogs and is closely related to *Dirofilaria immitis*. Infection with *D. immitis* can lead to immune-mediated glomerulonephritis characterized by immune complex deposition along the glomerular basement membrane, resulting in proteinuria and renal dysfunction. Reported histopathological changes include membranous glomerulonephritis with potential chronic progression to chronic interstitial nephritis, glomerulosclerosis, and amyloidosis. Despite the close relationship between these two Dirofilaria species, renal clinicopathological changes associated with *D. repens* infection have been only rarely investigated, and renal ultrastructural and immunofluorescence findings have not been described in naturally infected dogs. The objective of this study was to collect clinicopathological data and evaluate kidneys from dogs naturally infected with *D. repens* for structural abnormalities using light microscopy (LM), immunofluorescence (IF), and transmission electron microscopy (TEM).

**Results:**

Seventy-two shelter dogs from the university neutering program were screened for *D. repens* infection. Six infected dogs were identified, and renal biopsies were obtained during neutering. Serum urea, creatinine, and SDMA concentrations were measured, and comprehensive urinalysis was performed, including urinary protein-to-creatinine and albumin-to-creatinine ratios. None of the dogs had increased serum creatinine or SDMA; two of six dogs had mildly increased urea. Mean urine specific gravity was 1.029 ± 0.011, and urine sediment was unremarkable in all dogs. Two dogs were borderline proteinuric and one was proteinuric; the mean urine protein-to-creatinine ratio was 0.29 ± 0.15. Microalbuminuria was detected in one case (median: 0.001). Histopathology predominantly demonstrated podocyte injury with variable podocyte foot process effacement, without evidence supporting an immune complex–mediated glomerulopathy. Two dogs had mild focal and segmental glomerulosclerosis (FSGS). IF was available for two dogs and did not support immune complex–mediated disease, in agreement with TEM findings.

**Conclusions:**

In this cohort, dogs naturally infected with *D. repens* showed predominantly mild renal lesions characterized mainly by podocyte injury and, less frequently, focal segmental glomerulosclerosis. These findings differ from the immune-complex–dominant renal pathology commonly described in *D. immitis* infection and highlight the value of ultrastructural and immunofluorescence assessment for characterizing renal changes associated with *D. repens* infection.

**Supplementary Information:**

The online version contains supplementary material available at https://doi.org/10.1186/s12917-026-05535-3.

## Background

*Dirofilaria repens* is a widespread mosquito-borne filarial nematode that primarily causes subcutaneous dirofilariasis in dogs and cats and is a zoonotic agent responsible for pulmonary, subcutaneous, and ocular manifestations in humans [1]. In addition to domestic hosts, the parasite has been detected in wild carnivores, including foxes, golden jackals, wolves, and bears [2]. Across Europe, canine infection is particularly frequent in Mediterranean regions, with reported prevalence ranging from 0.2% to 30%, and the highest rates (17–49%) documented in Serbia [3]. In Hungary, the first autochthonous canine infection was reported in 1995 [4], and by 2017, the prevalence of *D. repens* infection among dogs with no travel history abroad reached 14.2% [5]. *D. repens* is closely related to *Dirofilaria immitis*; however, adult *D. repen*s typically inhabit subcutaneous and muscular connective tissues, whereas adult *D. immiti*s reside in the cardiopulmonary system and causes heartworm disease in a range of mammalian hosts, with domestic and wild canids most commonly affected. Both species release microfilariae into the bloodstream and share vectors among female culicid mosquitoes [1], with transmission reported for multiple mosquito species displaying different host preferences. One study indicates that the transmission of these parasites involves 12 mosquito species with different host preferences [6]. *D. immitis* adults may persist up to 7 years and microfilariae for up to 2 years [1], whereas *D. repens* adults and microfilariae may survive up to 4 years [7].

Renal involvement was observed in *D. immitis* infection, where immune complex formation along the glomerular capillary wall has long been linked to glomerulonephritis [8]. Many studies on experimentally infected beagle dogs have shown that the most common renal abnormality is membranoproliferative glomerulonephritis [9, 10]. Another research involving experimentally infected beagles found mild to moderate proteinuria, and histological examination of the kidneys revealed mild to severe diffuse chronic interstitial nephritis, along with generalized membranoproliferative glomerulonephritis [11]. One study in dogs with chronic *D. immitis* infection identified immune complex formation in the glomerular basement membrane [12], while additional studies confirmed ultrastructural evidence of filaria antibody immunocomplex nephropathy [13]. Others reported that dogs with circulating microfilariae have higher plasma D-dimer levels and an increased risk of renal microthrombosis [14]. Microfilariae, rather than adult worms, are mainly responsible for the described abnormalities. They contribute to renal damage through various mechanisms, including direct invasion of the glomerular capillaries. After treatment, degenerated microfilariae that partially or fully block glomerular capillaries were identified along with interstitial granulomas containing dead parasitic debris. These post-treatment lesions can be significant, with moderate-to-severe thickening of the glomerular basement membranes and widespread mesangial proliferation [15].

*D. repens* also causes microfilaremia, therefore it seems reasonable to assume that it can produce the same abnormalities as *D. immitis* microfilaria.

In a study conducted in Poland involving 188 dogs to assess the prevalence of *D. repens* infection, 12.7% tested positive. The presence of *D. repens* microfilariae was confirmed in parenchymal organs and the intestines during the post-mortem exam in two dogs. The kidneys exhibited membranoproliferative glomerulonephritis with crescents, focal glomerulosclerosis, chronic interstitial inflammation, and calcium deposits in the vessel walls, renal tubules, glomeruli, and interstitium. Small focal hemorrhages and thrombi were observed in the vessel lumens, and, in addition to calcium deposits, fibromatous necrosis was present in the vessel walls [16]. However, the presence of other organ changes in both dogs (endocarditis, right-sided heart failure, liver alterations) raised the possibility of concomitant diseases that may have caused or contributed to the renal changes. A comprehensive survey in Hungary found that dogs infected with *D. repens* had higher serum urea levels than the reference range. They also reported a case where the dog’s cause of death was renal fibrosis. In this case, the only identifiable abnormality was severe microfilaraemia caused by *D. repens* infection, leading them to hypothesize that immune-complex glomerulonephritis and multiple infarcts were responsible for the renal fibrosis [17]. To test this hypothesis, our research team conducted a previous study on naturally infected beagles and indeed observed significantly higher urinary albuminuria than in the control group [18]. The most compelling case report described a dog with a severe infection by *D. repens* (more than 300 adults! ) and a lesser infection by *D. immitis*. Light microscopic examination of the kidney showed extensive membranoproliferative glomerulonephritis affecting most of the glomeruli sampled, alongside fibromuscular atherosclerotic plaques, widespread fibrous and lymphohistiocytic interstitial nephritis, tubular mineralization, and epithelial desquamation. A large number of microfilariae were present at vascular, interstitial, and intratubular sites [19].

Besides the case reports mentioned, to our knowledge, no study has yet surveyed the microscopic renal lesions in dogs infected with *D. repens*, nor has any reported the findings of renal electron microscopy or immunofluorescence examinations in these dogs.

The objective of this study was to investigate renal biopsies from otherwise healthy, symptomless dogs infected with *D. repens* and to identify structural abnormalities associated with the infection using light microscopy, immunofluorescence, and electron microscopy. Additionally, the clinico-pathological data of these *D. repens*-infected dogs were documented.

## Methods

### Animals and inclusion criteria

The study protocol was approved by the Workplace Animal Welfare Committee of the University of Veterinary Medicine, Budapest. Following written informed consent obtained from the shelter director, a total of seventy-two clinically healthy shelter dogs of mixed breed were enrolled in the study between February 2021 and July 2021. The animals ranged in age from 12 to 81 months, with a median age of 24 months. Of these, 52 were female and 20 were male. The research was conducted in two phases: first, peripheral blood samples were collected from all dogs presented for routine ovariohysterectomy or castration as part of the standard pre-surgical evaluation; subsequently, renal biopsy samples were obtained only from dogs confirmed to be infected with *D. repens* and concurrently negative for all other infectious diseases included in the screening panel.

Dogs underwent two comprehensive clinical examinations, usually 1–3 weeks apart: the first before blood sampling and the second before neutering. Dogs were eligible if they were clinically healthy during both exams and met the following criteria: positive modified Knott test; negative *D. immitis* antigen ELISA and PCR; positive *D. repens* PCR; negative SNAP 4DX Plus test; and no significant changes in the listed hematological and biochemical parameters. Exclusion criteria included dogs that appeared clinically ill, or tested positive on *D. immitis* antigen ELISA, or on the SNAP 4DX Plus test, or showed major changes in the hematological and biochemical parameters listed above.

### Laboratory analysis

Five mL of whole blood was drawn from the cephalic vein using a 21 G needle, and the blood was divided into EDTA, citrate, and serum gel blood collection tubes. The blood samples were immediately transported to a veterinary laboratory, where all samples were screened, including a modified Knott test and a *D. immitis* antigen ELISA (Dirocheck ELISA kit, Zoetis). For the modified Knott test, 1 mL of homogenized EDTA-anticoagulated whole blood was combined with 9 mL of distilled water in a conical centrifuge tube and homogenized for at least 30 min. The mixture was then centrifuged at 4000 rpm for 10 min, the supernatant was decanted, and the sediment was stained with methylene blue. After vortex mixing, the sample was centrifuged again at 4000 rpm for an additional 10 min, and the final sediment was examined microscopically as a mounted slide preparation. Samples with a positive Knott test and a negative *D. immitis* antigen ELISA test were further examined. A duplex quantitative polymerase chain reaction (qPCR) was performed to distinguish between *D. immitis* and *D. repens* species.

After centrifugation of EDTA-anticoagulated peripheral blood samples, 200 µL of the buffy coat was collected, and DNA was extracted using the QIAGEN QIAamp DNA Mini Kit [cat. No. 51306] on the QIAcube robotic workstation (both Qiagen, Hilden, Germany) following the manufacturer’s instructions. Then we performed a duplex qPCR screening for the two *Dirofilaria* species. This protocol targets a 302 bp segment of the internal transcribed spacer 2 (ITS2) gene of *D. immitis* nuclear rDNA and a 209 bp segment of the mitochondrial cytochrome c oxidase subunit I (COX1) gene of *D. repens*, using primers described in an earlier publication [20]. Probes were labelled with FAM and Texas-Red dyes, respectively. In the qPCRs, forward and reverse primers for both targets were applied at the final concentration of 400nM and the probes at 100 nM. The reaction was carried out in a LightCycler^®^ 96 (Roche Diagnostics, Indianapolis, IN, USA) instrument using proprietary reagents. Briefly, the 10 µL final mixture contained five µL of LightCycler^®^ Probes Master (Roche, [cat. No. 06402682001]), one µL of each primer-probe mix, 0.5 µL of nuclease-free water, and 2.5 µL of the DNA extract. The thermal cycling protocol included a preincubation at 95 °C for 10 min, followed by 45 cycles of three-step amplification at 95 °C for 10 s, 55 °C for 15 s, and 60 °C for 30 s, then cooling.

Knott and *D. repens* PCR-positive dogs were considered infected with *D. repens* and tested further. Routine hematological (ADVIA 120, Siemens Healthcare GmbH, Erlangen, Germany), biochemical (Beckman Coulter AU480, Indianapolis, IN), and hemostasis (Sysmex CA-660, Siemens Healthcare GmbH, Erlangen, Germany) parameters from blood samples included the following parameters: complete blood count (CBC), reticulocyte count and related measurements, activated partial thromboplastin time (aPTT), prothrombin time (PT), total protein, albumin, globulin, alanine aminotransferase (ALT), aspartate aminotransferase (AST), alkaline phosphatase (ALP), gamma-glutamyl transferase (GGT), total bilirubin, cholesterol, urea, creatinine, potassium, sodium, chloride, phosphate, and Symmetric Dimethylarginine (SDMA). Reference intervals used for the routine hematological, biochemical and hemostasis paramters are the laboratory’s own reference ranges that were set up according to the ASVCP reference interval guidelines [21]. In addition, blood samples were analyzed using a qualitative ELISA to detect *Anaplasma phagocytophilum*, *Ehrlichia canis*, and *Borrelia burgdorferi* (SNAP 4DX Plus; IDEXX Laboratories Inc.*). Leishmania infantum* infection was not tested because this disease is not yet truly endemic in Hungary, and no clinical suspicion of the disease arose during the examinations. *Hepatozoon canis* and *Babesia sp*. infections were only checked from blood smears, as no clinical signs or laboratory changes compatible with the infections were observed.

Urine samples were collected from *D. repens*–infected dogs by cystocentesis and submitted to the same diagnostic laboratory (Praxislab Ltd., Budapest) for routine urinalysis, including urinary sediment examination, determination of the urine protein-to-creatinine ratio using an AU480 analyzer (Beckman Coulter, Indianapolis, IN), and measurement of the urine microalbumin-to-creatinine ratio using the same platform (Beckman Coulter AU480). To measure urinary albumin, a dedicated reagent kit (OSR6167, microalbumin, immunoturbidimetric) was used. Urinary creatinine levels were determined via an enzymatic assay, and total protein concentrations were measured using the pyrogallol red method (Diagnosticum 42 051). Proteinuria was defined as a urinary total protein-to-creatinine ratio (UPC), while albuminuria was indicated by a urinary microalbumin-to-creatinine ratio (UAC). Dogs were classified as borderline proteinuric if UPC > 0.2 and proteinuric if UPC > 0.5. Albuminuria was confirmed when UAC > 0.019 [22]. Routine urine analysis included measuring urine specific gravity with a refractometer, assessing urine color and transparency by the examiner from a predefined list, and testing urine pH, protein, hemoglobin/blood, glucose, ketones, nitrite, bilirubin, and urobilinogen using urine test strips analyzed by the LabuReader Plus 2 (77 Elektronika Kft, Budapest, Hungary). Sediment examination was conducted using the UriSed mini (77 Elektronika Kft, Budapest, Hungary), an automated device, with the examiner overseeing the results under a microscope.

### Renal biopsy and histology

All dogs enrolled in the study participated in the neutering program at the University of Veterinary Medicine, Budapest. During the neutering procedure, renal biopsies were collected from the *D. repens*-infected animals. The following anesthetic protocol was used during surgery. Premedication included midazolam 5 mg/ml at a dose of 0.05 ml/ kg body weight, ketamine 100 mg/ml at 0.005 ml/ kg body weight, and fentanyl 50 mcg/ml at 0.1 ml/ kg body weight, all administered intravenously. For induction, a 1% propofol injection at a dose of 0.5 ml/kg body weight was used, and anaesthesia was maintained with isoflurane inhalation. Postoperatively, dogs received meloxicam 5 mg/ml subcutaneously at a dose of 0.2 mg/ kg body weight. Male dogs underwent ultrasound-guided transcutaneous biopsy of the left kidney cortex in lateral recumbency following castration. The area was shaved and surgically prepared before obtaining the biopsy samples. Kidney biopsy samples from female dogs were collected directly from the kidneys after ovariectomy, before closing the abdominal incision. A16G semiautomatic single-use guillotine needle (Biomedical Srl, Firenze, Italy) was used to obtain the biopsy samples from the kidney cortex. After the renal biopsy, the dogs remained in the hospital for one day for further observation, and their abdomen was examined with ultrasound two hours and 12 h later to rule out intra-abdominal bleeding after the biopsy. No complications were observed in any of the biopsied dogs. Renal biopsy specimens were placed in formalin for light microscopy (LM), 3% glutaraldehyde for transmission electron microscopy (TEM), and Michel’s solution for immunofluorescence (IF) and were subsequently submitted to the European Veterinary Renal Pathology Service for diagnostic evaluation (https://evrps.mylav.net/) [23]. LM sections were cut at 3 μm and stained with hematoxylin and eosin, periodic acid–Schiff, Masson’s trichrome, and periodic acid-methenamine silver. For TEM, tissues wer processed using standard protocols as previously described. For IF the follwing antibodies were used: anti-IgG, anti-IgM, anti-IgA, and anti-C3 [24]. The evaluation and classification of renal lesions followed diagnostic criteria. These were established by the WSAVA Renal Standardization Study Group. TEM and IF served as the gold standards for detecting and phenotyping immune complex deposits. Based on the morphological findings, the shelter was provided with guidance on recommended therapeutic management and expected prognosis.

### Statistics

Data were organized in Excel sheets and analyzed using the Numiqo Online Statistics Calculator (numiqo e.U. Graz, Austria. URL https://numiqo.com). The data distribution was assessed using the Shapiro-Wilk test. Normally distributed data are presented as mean ± SD, while non-normally distributed data are shown as median and interquartile range.

## Results

Of the 72 dogs included in the study, 12 were positive on the Knott test, and among them, six had negative *D. immitis* ELISA results. All six samples tested positive for *D. repens* by PCR and were included in the study. From all six dogs, a kidney biopsy and a urine sample were obtained. These six dogs were between 42 and 66 months old (median, 57 months); four were male, and two were female. In Table 1, the most important biochemical parameters, serology results, and urine sample findings are reported. One dog exhibited mild hypoproteinemia and another mild hyperproteinemia (total protein, 66 ± 11 g/L; mean ± SD). Albumin concentrations were within the reference range in all dogs (31 ± 4 g/L). Mild azotemia, reflected by increased urea, was detected in two dogs (6.8 and 14.2 mmol/L), whereas the overall median urea concentration was 5.4 mmol/L (IQR, 2.0); serum creatinine values were within the reference range in all dogs (60 ± 19 µmol/L). Mean serum phosphorus was 1.5 ± 0.1 mmol/L and SDMA was 8 ± 1 µg/dL.

**Table 1:** Selected laboratory results of the six dogs infected with *D. repens*

Dog	Reference interval	1	2	3	4	5	6
Age		66 months	66 months	42 months	66 months	48 months	48 months
Sex		male	female	male	male	female	male
Total protein	55–75 gram/litre	52	59	72	82	62	66
Albumin	25.0–41.0 gram/litre	25.7	28.7	35.2	34.3	30.9	29.5
Urea	2.5–6.7 millimol/litre	4.1	3.9	6.8	14.2	5.1	5.6
Creatinine	20–150 micromol/litre	38	50	67	92	62	53
Phosphorus	0.8–1.6 millimol/litre	1.6	1.4	1.7	1.5	1.5	1.3
SDMA	0–14 µg/decilitre	9.5	7.8	6.7	9.4	8.7	6.4
Urine specific gravity	1030–1090	1010	1034	1040	1036	1024	1030
Urine protein		negative	+	+	+	negative	+
Sediment red blood cell	− 5	0	0	0–2	0	0	0
Sediment white blood cell	− 5	0	0	0	0–1	0	0
Urinary total protein-to-creatinine ratio	− 0.50	0.19	0.4	0.32	0.51	0.15	0.17
Urinary albumin-to creatinine-ratio	0-0.019	0	0.027	0.005	0.001	0.003	0

**Fig. 1:**
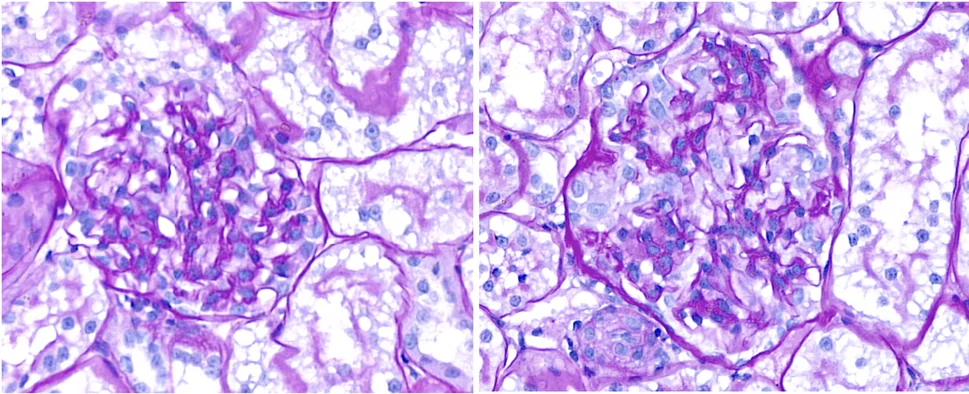
Podocytopathy with Periodic acid–Schiff (PAS) stain Two glomeruli showing moderate podocytopathy, characterized by mild-to-moderate glomerular tuft changes with preserved overall architecture and no evident sclerosis

**Fig. 2:**
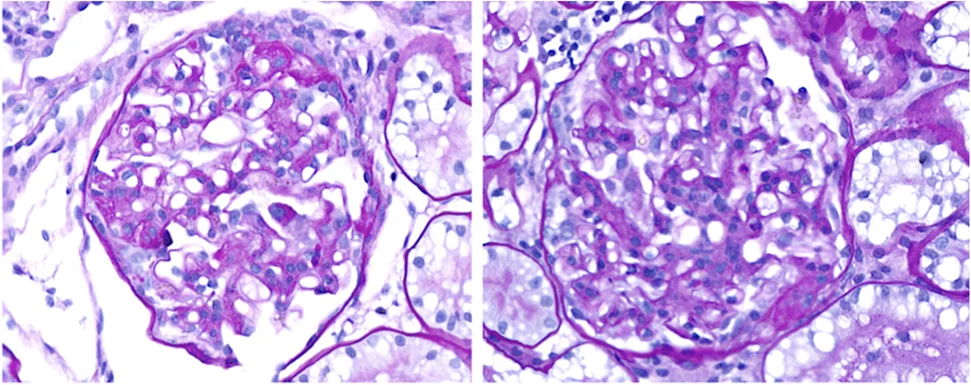
Focal segmental glomerulosclerosis with Periodic acid–Schiff (PAS) stain Two glomeruli showing mild focal segmental glomerulosclerosis (FSGS), characterized by segmental sclerosis and increased mesangial matrix affecting a portion of the glomerular tuft

The mean urine specific gravity was 1.029 ± 0.011, and all dogs had a negative urine sediment. Three dogs were not proteinuric; two were borderline proteinuric; and one had proteinuria. The mean total protein-to-creatinine ratio was 0.29 ± 0.15. Microalbuminuria was detected in one sample (median: 0.001). All six renal biopsy specimens from D. repens–infected dogs were sufficient for light microscopy (LM) and transmission electron microscopy (TEM). However, immunofluorescence (IF) could only be performed in two dogs (dogs 5 and 6) because the other samples lacked available glomeruli. In Table 2 the results of the histological examinations are reported. Overall, the predominant lesion pattern was podocyte injury (Fig. 1) with variable podocyte foot process effacement, without a convincing immune-complex–mediated glomerulopathy. Electron-dense deposits were not identified in any case; endocapillary hypercellularity was not a feature, and only sparse nonspecific intramembranous electron densities, with limited segmental subendothelial basement membrane remodeling, were observed focally in two dogs (dogs 2 and 6). These two dogs were most consistent with podocytopathy on TEM, characterized by extensive (dog 2) or focal (dog 6) foot process effacement with podocyte vacuolization and moderate-to-marked microvillous transformation, accompanied by mild mesangial matrix expansion and mild segmental subendothelial glomerular basement membrane thickening/lucency. IF examination in dog 6 was negative for IgG, IgM, and C3 (IgA was not assessable due to lack of glomeruli). Two dogs (1 and 3) showed focal and segmental glomerulosclerosis (Fig. 2) on LM (mild in dog 1; early-stage with hyalinosis in dog 3) with only focal-to-mild effacement on TEM; dog 3 additionally had multifocal mild-to-moderate lymphoplasmacytic interstitial nephritis. Tubulointerstitial lesions were variably present across the series, including mild patchy tubular degeneration/atrophy (dog 1), mild interstitial nephritis with fibrosis and tubular atrophy (dog 2), and multifocal mild lymphohistiocytic interstitial nephritis with tubular degeneration/atrophy and mild-to-moderate podocyte injury (dog 4). Dog 5 had mild mesangial sclerosis with mild tubular atrophy and minimal ultrastructural change, with IF showing equivocal IgG and weak segmental splotchy C3 (1+) labeling (IgM and IgA negative).

**Table 2:** Results of light microscopy (LM), transmission electron microscopy (TEM), and immunofluorescent (IF) examinations of renal biopsy samples from the six dogs infected with *D. repens*

Dog	LM result	IF result	TEM result	Final diagnosis
1	Suspect of mesangio-proliferative glomerulonephritis; mild patchy tubular degeneration and atrophy.	-	Mild segmental podocyte foot process effacement. Glomerular capillary walls are of normal thickness with minimal increased thickness of the paramesangium. Electron-dense deposits are not identified.	Mild focal and segmental glomerulosclerosis.
2	Mild podocytopathy and mesangio-sclerosis; mild interstitial nephritis, fibrosis, and tubular atrophy.	-	Widespread, extensive foot process effacement and vacuolization of podocytes. Podocytes fill the Bowman’s space; there is moderate to marked microvillous transformation. Mild increase of the mesangial matrix. Mild, segmental thickening and lucency of the subendothelial (luminal) glomerular basement membrane with focal remodeling associated with the endothelial cell. Few speckled electron densities are scattered within the glomerular basement membrane.	Evidence of widespread podocyte injury, and thus a diagnosis of podocytopathy is most appropriate.
3	Focal segmental glomerulosclerosis and hyalinosis, multifocal mild to moderate lymphoplasmacytic interstitial nephritis.	-	Focal podocyte foot process effacement, with most of the foot processes remaining intact. Focal thickening and wrinkling of the glomerular basement membranes are associated with rarefaction. In a few spots, this is compatible with immune complexes reabsorption in the subendothelial space. Rare small electron densities are scattered within the expanded glomerular basement membrane; however, electron-dense deposits consistent with immune complexes are absent.	Focal and segmental glomerulosclerosis at an early stage.
4	Multifocal mild lymphohistiocytic interstitial nephritis with tubular degeneration and atrophy; mild to moderate podocyte injury	-	Mild segmental podocyte foot process effacement, but most podocytes maintain intact foot processes. Glomerular capillary walls are of normal thickness with minimal increased thickness of the paramesangium. Electron-dense deposits are not identified within mesangial zones or along capillary walls, and hypercellularity is not present.	Multifocal mild lymphohistiocytic interstitial nephritis with tubular degeneration and atrophy; mild to moderate podocyte injury
5	Mild mesangial sclerosis associated with mild tubular atrophy	● IgG: +/- ● IgM: - ● C3: Segmental weak (1+) splotchy labeling ●IgA: -	Small segments of podocyte foot process effacement, but most foot processes remain intact. In one lobule, there is mild wrinkling and multi-lamination of the glomerular basement membrane, but other capillary loops are within normal limits. Electron-dense deposits are not identified along capillary walls or in the mesangial zones.	Mild mesangial sclerosis associated with mild tubular atrophy
6	Renal sample within normal limits	● IgG: - ● IgM: - ● C3: - ● IgA: no glomeruli available	Focal foot process effacement and vacuolization of podocytes. Moderate to marked microvillous transformation. Mild increase in the mesangial matrix. Mild, segmental thickening and lucency of the subendothelial (luminal) glomerular basement membrane with focal remodeling associated with the endothelial cell. Few speckled electron densities are scattered within the glomerular basement membrane, but otherwise, outer contours are smooth, and endocapillary hypercellularity is not a feature.	Evidence of widespread podocyte injury, and thus a diagnosis of podocytopathy is most appropriate.

## Discussion

To the best of our knowledge, this study is the first to report on renal histomorphology using LM, IF, and TEM in dogs with naturally occurring *D. repens* mono-infection. Using this combined approach, mild-to-moderate podocyte injury emerged as the predominant lesion pattern: three of six dogs (50%) showed changes consistent with podocytopathy, while two of six (33%) had mild focal and segmental glomerulosclerosis (FSGS). In addition, one dog showed mild mesangial sclerosis with mild tubular atrophy. From a clinicopathological standpoint, these diagnoses are relevant because podocytopathies represent potentially reversible podocyte injury, whereas FSGS is typically irreversible and may progress to chronic kidney disease; nevertheless, therapeutic management overlaps substantially and is centered on antiproteinuric and renoprotective strategies (e.g., optimization of renin–angiotensin system inhibition, dietary management, and blood pressure control), with prognosis being comparatively less favorable in FSGS due to its progressive nature [25, 26].

Notably, the renal phenotype observed here differed from the canonical lesions described in *D. immitis*–associated nephropathy. In heartworm-infected dogs, thickening and vacuolation of the glomerular basement membrane (GBM), mesangial hypercellularity/proliferation, podocyte foot process effacement, and electron-dense deposits within the GBM and mesangium are frequently reported, consistent with immune-complex-mediated membranoproliferative glomerulonephritis [10]. In contrast, there was no compelling ultrastructural evidence of immune-mediated glomerulonephritis in the present series. Electron-dense immune-type deposits were not identified in any dog, and endocapillary hypercellularity was not a feature. In one case (dog 3), in which the final diagnosis was early-stage FSGS, focal ultrastructural changes compatible with prior subendothelial immune-complex reabsorption were observed, raising the possibility of a regressed or resolving immune-complex process; however, in the absence of demonstrable deposits, these findings were insufficient to support a diagnosis of immune-complex glomerulonephritis.

Interpretation of immune labeling was further constrained by limited IF availability (2/6 dogs): dog 5 showed only mild IgG and C3 positivity with negative results for other markers, and dog 6 was negative for IgG, IgM, and C3 (IgA not assessable due to lack of glomeruli). Overall, the IF results did not support immune-complex–mediated disease and were consistent with the TEM findings.

The diagnostic contribution of TEM was substantial in this study. Agreement between LM and TEM for morphological diagnosis and disease category was only moderate, consistent with previously reported observations that ultrastructural assessment can refine or alter lesion categorization in canine renal pathology [23]. In practical terms, TEM was essential to confirm the dominant pattern of podocyte injury, to exclude electron-dense deposits, and to distinguish early or subtle glomerular disease from more advanced, irreversible lesions.

Clinico-pathological data supported the interpretation of predominantly mild renal involvement. Serum albumin, creatinine, and SDMA were within reference intervals in all dogs, consistent with preserved global renal function, whereas urea was mildly increased in two dogs, which may reflect a prerenal component or very early renal involvement not yet expressed as creatinine/SDMA elevation. These findings are consistent with the broader concept that azotemia may be absent in early-to-moderate filarial infection despite the presence of microscopic glomerular lesions. Proteinuria (UPC > 0.2) was present in three of six dogs (50%), overt proteinuria (> 0.5) in one dog (16%), and albuminuria in one dog (16%). This pattern is broadly comparable with previous observations in naturally infected dogs, although overt proteinuria and albuminuria occurred at lower frequencies in the present series than reported by Falus et al. (2023) [18]. In contrast, experimental *D. immitis* infection has been associated with higher prevalences of proteinuria and albuminuria [27], emphasizing that the renal functional signature of *D. repens* infection may be milder or more heterogeneous. Importantly, proteinuria remains a clinically meaningful early marker of glomerular injury and a prognostic indicator, as persistent protein loss can promote progressive glomerular and tubulointerstitial damage [28].

The prevalence of *D. repens* infection in the shelter dog population included in the sample was 8.3%, which is close to the national estimates previously published in Hungary (without *D. immitis* co-infections) and comparable to the latest survey data on Italian shelter dogs [5, 29]. Several factors may account for the absence of a consistent immune-complex glomerulonephritis pattern and for the generally mild-to-moderate lesions observed. First, *D. repens* may provoke a milder systemic immune response than *D. immitis*, leading to a lower likelihood of immune complex deposition and membranoproliferative changes. Second, because infections were naturally acquired, neither the onset nor the duration of infection could be determined; a shorter infection course could plausibly precede the development of the more advanced glomerular alterations documented in experimental heartworm studies [10]. In *D. immitis* infection, lesion severity correlates with infection parameters such as duration (> 1 year), microfilaremia intensity, and adult worm burden, with higher burdens associated with more prominent GBM thickening, more extensive foot process effacement, and greater mesangial proliferation [10]. Third, the small sample size limits the ability to infer the full range of renal pathology that may occur in naturally infected populations and decreases the chance of detecting rare but severe phenotypes.

Despite these limitations, the observation of FSGS in a subset of dogs may be of clinical and biological interest, suggesting a possible association between *D. repens* infection and renal lesions that partially overlap with those described in other filarial infections, although the dominant pattern differs from classic immune-complex membranoproliferative glomerulonephritis. These findings should be interpreted with caution, as they indicate that histopathological renal changes can be present in dogs naturally infected with *D. repens* even in the absence of overt biochemical evidence of kidney dysfunction. Larger studies of naturally infected dogs, ideally supported by longitudinal sampling and standardized assessment of microfilaremia and worm burden, as well as controlled experimental infections, are required to clarify causality, lesion development, and prognostic significance.

## Conclusions

In summary, dogs naturally infected with *D. repens* in this cohort showed mainly mild to moderate kidney damage, mainly involving podocyte injury and, less often, FSGS. This differs from the immune-complex–dominant kidney damage often seen in *D. immitis* infection.

## Supplementary Information

Supplementary Material 1.

## Data Availability

Availability of data and materials supporting the findings of this study is available upon reasonable request from the corresponding author.

## Abbreviations

ALP: alkaline phosphatase
ALT: alanine aminotransferase
aPTT: activated partial thromboplastin time
AST: aspartate aminotransferase
CBC: complete blood count
D. immitis: *Dirofilaria immitis*
D. repens: *Dirofilaria repens*
FSGS: focal segmental glomerulosclerosis
GBM: glomerular basement membrane
GGT: gamma-glutamyl transferase
IF: immunofluorescence microscopy
LM: light microscopy
PT: prothrombin time
qPCR: quantitative polymerase chain reaction
SDMA: Symmetric Dimethylarginine
TEM: transmission electron microscopy
TP: total protein
UAC: urinary microalbumin-to-creatinine ratio
UPC: urinary total protein-to-creatinine ratio
