# Supplementary material for: Clinico-pathological and renal morphological findings in dogs naturally infected with Dirofilaria repens
Source: BMC Vet Res. 2026 May 2;22:361. doi: 10.1186/s12917-026-05535-3 (PMC13285513; doi:10.1186/s12917-026-05535-3)
Supplement: Supplementary file 1 — Supplementary Material 1. [file 12917_2026_5535_MOESM1_ESM.docx]

|  | **Dog** | **1** | **2** | **3** | **4** | **5** | **6** |
| --- | --- | --- | --- | --- | --- | --- | --- |
| **Laboratory Parameter** | **Reference range** |  |  |  |  |  |  |
| **Red blood cell** | **5,50 - 8,50 T/L** | 7.37 | 8.28 | 6.78 | 7.26 | 7.22 | 6.53 |
| **Hemoglobin** | **120 – 180 g/L** | 173 | 193 | 166 | 172 | 164 | 151 |
| **Hematocrit** | **38.0 - 57.0 %** | 49.4 | 57.7 | 47.3 | 50.1 | 48.8 | 43.8 |
| **MCV** | **61 – 80 fL** | 67 | 70 | 70 | 69 | 68 | 67 |
| **MCH** | **20.0 - 26.0 pg** | 23.5 | 23.3 | 24.6 | 23.6 | 22.7 | 23.1 |
| **MCHC** | **300 – 360 g/L** | 351 | 335 | 352 | 342 | 335 | 345 |
| **RDW** | **12.0 - 13.2** | 13.2 | 12.5 | 14.2 | 13 | 13.6 | 13.6 |
| **Platelet** | **150 – 450 G/L** | 311 | 310 | 337 | 292 | 283 | 351 |
| **MPV** | **8.5 - 14.5 fL** | 10.9 | 8.3 | 11.2 | 10.6 | 9.9 | 11.5 |
| **White blood cell** | **6.0 - 15.0 G/L** | 6.8 | 11.1 | 11.3 | 8.3 | 12.1 | 11.4 |
| **Neutrofil granulocyte** | **4.3 - 9.0 G/L** | 5 | 5.7 | 5.8 | 4.3 | 6.5 | 7.7 |
| **Lymphocyte** | **0.50 - 4.50 G/L** | 1.08 | 4.19 | 3.6 | 2.95 | 3.67 | 2.35 |
| **Monocyte** | **0.25 - 1.00 G/L** | 0.31 | 0.41 | 0.34 | 0.22 | 0.39 | 0.69 |
| **Eosinophil granulocyte** | **0.10 - 1.20 G/L** | 0.4 | 0.79 | 1.59 | 0.78 | 1.45 | 0.67 |
| **Basophil granulocyte** | **0.01 - 0.08 G/L** | 0.02 | 0.03 | 0.02 | 0.02 | 0.09 | 0.03 |
| **LUC** | **0.03 - 0.58 G/L** | 0.01 | 0.02 | 0.01 | 0.02 | 0.01 | 0.01 |
| **Reticulocyte** | **<60 G/L** | 64.2 | 78.3 | 45.7 | 50.1 | 143.3 | 43.3 |
| **MCVR** | **81.00 - 97.00 fL** | 89.6 | 89 | 89.4 | 89.1 | 86.5 | 86.9 |
| **LRTC** | **68.0 - 647.0** | 267 | 321 | 215 | 233 | 472 | 249 |
| **LRT%** | **65.0 - 91.0** | 59.5 | 63.3 | 67 | 67.3 | 48.7 | 80.6 |
| **MRT** | **6.40 - 260.0** | 111 | 109 | 55 | 48 | 215 | 37 |
| **MRT%** |  | 24.7 | 21.5 | 17.1 | 13.9 | 22.2 | 12 |
| **HRT** |  | 71 | 77 | 51 | 65 | 283 | 23 |
| **HRT%** | **4.30 - 10.00** | 16 | 15 | 16 | 19 | 29 | 7 |
| **modified KNOTT test** |  | positive | positive | positive | positive | positive | positive |
|  |  |  |  |  |  |  |  |
| **Prothrombin time** | **6.7 - 9,2 sec** | 8.2 | 9.3 | 8.2 | 7 | 7.1 | 7.6 |
| **Activated partial thromboplastine time** | **9.2-13.1 sec** | 9.3 | 11.3 | 9.4 | 12.3 | 10.2 | 9.9 |
| **Total protein** | **55 – 75 g/L** | 52 | 59 | 72 | 82 | 62 | 66 |
| **Albumin** | **25.0 - 41.0 g/L** | 25.7 | 28.7 | 35.2 | 34.3 | 30.9 | 29.5 |
| **Globulin** | **20.0 - 45.0 g/L** | 26.3 | 30.3 | 36.8 | 47.7 | 31.1 | 36.5 |
| **ALT** | **5 – 60 U/L** | 34 | 41 | 55 | 56 | 38 | 59 |
| **AST** | **10 – 50 U/L** | 40 | 31 | 42 | 42 | 32 | 44 |
| **ALP** | **- 280 U/L** | 120 | 84 | 109 | 86 | 73 | 136 |
| **GGT** | **- 9 U/L** | 5 | 6 | 5 | 7 | 2 | 8 |
| **Total bilirubin** | **0.1 - 5.1 umol/L** | 4.3 | 1.1 | 2.4 | 3.2 | 2.5 | 4.5 |
| **Cholesterol** | **3.2 - 6.2 mmol/L** | 2 | 5.1 | 6.9 | 5.9 | 5.5 | 4.9 |
| **Urea** | **2.5 - 6.7 mmol/L** | 4.1 | 3.9 | 6.8 | 14.2 | 5.1 | 5.6 |
| **Creatinine** | **20 – 150 umol/L** | 38 | 50 | 67 | 92 | 62 | 53 |
| **Sodium** | **135 – 155 mmol/L** | 145 | 148 | 147 | 141 | 148 | 144 |
| **Potessium** | **3.60 - 5.60 mmol/L** | 5.02 | 4.79 | 4.84 | 4.52 | 5.11 | 4.84 |
| **Chloride** | **100 – 116 mmol/l** | 107 | 110 | 108 | 106 | 110 | 106 |
| **Phosphate** | **0.8 - 1.6 mmol/L** | 1.6 | 1.4 | 1.7 | 1.5 | 1.5 | 1.3 |
| **SDMA** | **0-14 ug/dL** | 9.50 | 7.80 | 6.70 | 9.40 | 8.70 | 6.40 |
| **Dirofilaria immitis ELISA (Dirocheck)** |  | negative | negative | negative | negative | negative | negative |
| **Dirofilaria immitis RT-PCR** |  | negative | negative | negative | negative | negative | negative |
| **Dirofilaria repens RT-PCR** |  | positive | positive | positive | positive | positive | positive |
| **IDEXX 4 DX Anaplasma spp.** |  | negative | negative | negative | negative | negative | negative |
| **IDEXX 4 DX Ehrlichia spp.** |  | negative | negative | negative | negative | negative | negative |
| **IDEXX 4 DX Dirofilaria Immitis** |  | negative | negative | negative | negative | negative | negative |
| **IDEXX 4 DX Borrelia Burgdorferi C6 antibodies** |  | negative | negative | negative | negative | negative | negative |
| **Hemolysis** |  | Normal | Normal | Normal | Normal | Normal | + |
| **Icterus** |  | Normal | Normal | Normal | Normal | Normal | Normal |
| **Lipemia** |  | Normal | Normal | Normal | Normal | Normal | Normal |
|  |  |  |  |  |  |  |  |
| **Urine colour** |  | pale yellow | pale yellow | dark yellow | dark yellow | dark yellow | dark yellow |
| **Urine transparency** |  | opalescent | opalescent | opalescent | opalescent | transparent | opalescent |
| **Urine specific garvity** | **1030 - 1090** | 1010 | 1034 | 1040 | 1036 | 1024 | 1030 |
| **Urine Ph** |  | 5.5 | 6.5 | 6.5 | 6 | 5 | 6.5 |
| **Urine protein** |  | negative | + | + | + | negative | + |
| **Urine hemoglobin** |  | negative | negative | negative | + | negative | negative |
| **Urine glucose** |  | negative | negative | negative | negative | negative | negative |
| **Urine keton** |  | negative | negative | negative | pigmenturia | negative | negative |
| **Urine nitrate** |  | negative | negative | negative | negative | negative | negative |
| **Urine bilirubin** |  | negative | negative | + | + | + | negative |
| **Urine urobilinogen** |  | negative | negative | negative | negative | negative | negative |
| **Sediment red blood cell** | **- 5** | 0 | 0 | 0-2 | 0 | 0 | 0 |
| **Sediment white blood cell** | **- 5** | 0 | 0 | 0 | 0-1 | 0 | 0 |
| **Sediment epithelial cell** |  | 0 | 0 | 0 | 0 | 0 | 0 |
| **Sediment cristal** |  | 0 | 0 | 0 | 0 | 0 | 1-2 bilirubin |
| **Other** |  | sperm | 0 | sperm | sperm | 0 | sperm |
| **Urine total protein-to- creatinine ratio** | **- 0.50** | 0.19 | 0.4 | 0.32 | 0.51 | 0.15 | 0.17 |
| **Urinary albumine-to creatinine ratio** | **0-0,019** | 0 | 0.027 | 0.005 | 0.001 | 0.003 | 0 |

Reference intervals used for the routine hematological, biochemical and hemostasis paramters are the laboratory’s own reference ranges that were set up according to the ASVCP reference interval guidelines [21].
